# Overexpression of Id-1 is significantly associated with tumour angiogenesis in human pancreas cancers

**DOI:** 10.1038/sj.bjc.6601684

**Published:** 2004-02-24

**Authors:** K T Lee, Y W Lee, J K Lee, S H Choi, J C Rhee, S S Paik, G Kong

**Affiliations:** 1 Departments of Internal Medicine and General Surgery, Samsung Medical Center, Sungkyunkwan University, School of Medicine, Seoul, Korea; 2 Department of Pathology, College of Medicine, Hanyang University, Seoul, Korea

**Keywords:** Id-1 overexpression, tumour angiogenesis, pancreas cancer, prognosis, survival rate

## Abstract

It has been suggested that Id-1 has a critical role in the tumour progression and aggressiveness of several human cancers. However, the clinicopathological and biological significance of Id-1 overexpression remains unclear in human primary cancer. To investigate the association between Id-1 expression and cell proliferation or tumour angiogenesis, we examined the cell cycle kinetic indices (the proliferation and apoptotic indices, PI and AI) and intratumoral microvessel density (MVD) in 65 human pancreatic cancers. We also investigated the relationship between its expression and various clinicopathological factors to determine the clinical significance of Id-1 overexpression. Out of a total 65 cases, 32 (49.3%) showed overexpression of Id-1 *vs* normal tissues. Id-1 expression was found to be significantly associated with MVD (*P*=0.002). In further analysis of subgroups with higher and lower Id-1 expression, tumours with higher Id-1 expression (scores 4 and 5) showed significantly higher MVD than tumours with lower expression of Id-1 (scores 2 and 3) (111.18±57.14 *vs* 64.13±28.19, *P*<0.001). However, no significant association was found between Id-1 overexpression and patient survival rate. No significant association was also found between Id-1 expression and cell cycle kinetic indices (PI or AI) in pancreatic cancer. Moreover, the overexpression of Id-1 protein was not correlated with any significant clinicopathologic factors. These findings indicate that Id-1 overexpression is closely related with tumour angiogenesis and a higher density of intratumoral vessel, but that it is not associated with a poorer prognosis of survival or a higher cell proliferative potential in human pancreatic cancer.

Id proteins (inhibitors of differentiation or DNA binding) are members of a family of basic helix–loop–helix (bHLH) transcription factors lacking the DNA-binding domain. Therefore, they act as dominant-negative regulators of basic HLH proteins by forming transcriptionally inactive Id-bHLH protein complexes (Riechmann *et al*, 1994; Riechmann and Sablitzky, 1995). Id proteins play key roles in the regulation of cell differentiation during neurogenesis, lymphoiesis and angiogenesis. Their functions also include the promotion of cell growth and cell cycle progression, and apoptotic induction (Lyden *et al*, 1999; Norton, 2000).

Recently, several reports have shown that Id-1 protein can induce cell proliferation and increase DNA synthesis, and that it can immortalise mammalian cells in cooperation with other oncogenes (Norton and Atherton, 1998; Alani *et al*, 1999). Id-1 overexpression has been found in several types of primary human cancers including breast, pancreatic, prostate, melanoma and cervical cancers (Maruyama *et al*, 1999; Langlands *et al*, 2000; Lin *et al*, 2000; Polsky *et al*, 2001; Schindl *et al*, 2001; Ouyang *et al*, 2002a; Wang *et al*, 2002; Schindl *et al*, 2003; Schoppmann *et al*, 2003). Moreover, the ectopic expression of Id-1 increases the proliferation, migration, invasion and metastasis of breast cancer cells (Desprez *et al*, 1998; Lin *et al*, 2000). In addition, Id-1 seems to be an essential factor in the promotion of G1/S cell cycle transition in certain cancers by inactivating of p16 and increasing of CDK4 activity/RB (Polsky *et al*, 2001; Ouyang *et al*, 2002b; Singh *et al*, 2002). These lines of evidence strongly suggest that Id proteins play important roles not only in tumorigenesis and in tumour progression.

Id-1 and Id-3 may induce angiogenesis by regulating the growth and invasion of endothelial cells. They are also required for proper angiogenesis in the neuroectoderm during mouse development (Lyden *et al*, 1999; Norton, 2000). Moreover, the downregulation of these proteins results in an angiogenic defect in growth and metastasis of tumour xenografts (Lyden *et al*, 1999). Id-1 can also induce the expression of a novel metalloproteinase of 120 kDa in breast epithelial cells (Desprez *et al*, 1998). These studies indicate that Id-1 has a potential role in the aggressiveness of malignant tumour and in formation of tumour angiogenesis.

Pancreatic cancer is one of the most aggressive malignant tumours and is associated with the activation of various oncogenes such as Ki-ras and the inactivation of tumour suppressor genes, for example, p16 and p53 (Korc, 1998). Recently, Maruyama *et al* demonstrated that Id-1 is significantly overexpressed in pancreatic cancer rather than chronic pancreatitis, suggesting that Id-1 protein may be associated with the enhanced proliferative potential of pancreatic cancer cells. However, no evidence is available upon the prognostic implications of Id-1 in human pancreatic cancers. Therefore, we undertook to examine Id-1 overexpression in human pancreatic cancers to determine its role as a poor prognostic factor. Furthermore, we investigated its association with intratumoral microvessel density, cell proliferation and apoptosis to clarify its role in the proliferation potential of tumour cells and in the induction of tumour angiogenesis. Our results show that Id-1 overexpression is significantly related with tumour angiogenesis, higher density of intratumoral vessel, but not associated with a poorer prognosis or a higher proliferative potential in human pancreatic cancers. We provide the first evidence to clarify the association between Id-1 overexpression and tumour angiogenesis in human primary cancers and suggest that Id-1 protein may be an important new target molecule for antiangiogenic drugs in cancer treatment.

## MATERIALS AND METHODS

### Patients and tissue samples

In all, 65 pancreatic ductal adenocarcinoma cases that successfully underwent resection at Samsung Medical Center (Seoul, Korea) between January 1995 and September 1999 were enroled in this retrospective study. The mean follow-up interval was 12.6 months (range, 1–66.2 months). In total, 51 (78.5%) patients died and 14 (21.5%) patients remained alive during the follow-up period. Haematoxylin–eosin stains were reviewed by two pathologists and the most representative paraffin-embedded blocks were chosen for further analysis. Histological grade was evaluated according to the established AJCC criteria.

The clinicopathologic characteristics of the 65 patients, which included age, bilirubin level, tumour marker (CA 19–9), location, size, AJCC stage, differentiation, distant metastasis and survival, were evaluated. The median age of the patients at the time of operation was 60.4 years (range: 40–79 years). Tumours were classified according to the location and AJCC stage based on data obtained by clinical examination, imaging methods, operation records and surgical specimens.

### Immunohistochemical staining

Immunohistochemical staining for Id-1, Ki-67 and CD-34 was performed by using avidin–biotin peroxidase complex method with aminoethylcarbamazole (AEC) as chromogen using LSAB kit (DAKO, Carpinteria, CA, USA). Paraffin-embedded tissue blocks, which included tumour and normal pancreatic tissues, were sectioned into 5 *μ*m. The slides were deparaffinised using xylene, and rehydrated using graded alcohols. The slides were then incubated in 3% hydrogen peroxide for 10 min to block endogenous peroxidase activity. Primary antibodies of Id-1 (Santa Cruz, Santa Cruz, CA, USA), Ki-67 (Immunotech, France), and CD-34 (Immunotech, France) were used at a dilution of 1 : 50. Then, secondary biotinylated antibody and avidin–biotin complex reagent were applied and the sections were counterstained with haematoxylin and mounted. Because Id-1 is strongly expressed in the smooth muscle cells of vessels, they were used as an internal positive control in each section (Dorai and Sampath, 2001). The Id-1 immunoreactivity shown by smooth muscle cells of blood vessels was also used as a reference for strong immunoreactivity. As a negative control, sections were incubated with Tris-buffered saline containing 2% rabbit serum and 1% bovine serum albumin instead of the primary antibody. The Id-1 immunoreactivity was graded based on the intensity and the number of positive cells, as described in the previous study (Maruyama *et al*, 1999). The percentage of positive cancer cells was classified into four groups: 0, no tumour cells exhibiting immunoreactivity; 1, <33% immunoreactivity; 2, 33–67% immunoreactivity; 3, >67% immunoreactivity. The immunohistochemical signal intensities were also classified into four groups: 0, no immunoreactivity; 1, weak immunoreactivity; 2, moderate immunoreactivity; 3, strong immunoreactivity. Finally, we summed the area and the intensity scores. The Ki-67 labelling index (Proliferation Index; PI) was determined by counting the percentage of positive cells among 1000 tumour cells in 10 random regions in × 400 fields. Intratumoral microvessel density (MVD) was recorded by counting CD-34-positive vessels in the highest vascularised area in four × 200 fields. Blood vessels with lumen diameter larger than approximately eight red blood cells were excluded.

### Detection of apoptotic cells

Cells undergoing programmed cell death (apoptosis) were detected by *in situ* specific labelling of nuclear DNA fragmentation method (TUNEL method) using ApopTag *In situ* apoptosis detection kit (Oncor, Gaithersburg, MD, USA). Paraffin-embedded thin sections (5 *μ*m) were deparaffinised and digested with 20 *μ*g ml^−1^ proteinase K (DAKO, Carpinteria, CA, USA). Endogenous peroxidase activity was blocked with the treatment of 2.0% hydrogen peroxide in phosphate-buffered saline (PBS) for 5 min. The sections were then incubated with working strength TdT enzyme in a humidified chamber at 37°C for 1 h, rinsed with working strength stop/wash buffer, and incubated with anti-digoxigenin peroxidase for 30 min. Diaminobenzidine (DAKO, Carpinteria, CA, USA) was applied for colour development. The apoptotic index (AI) was calculated by counting the percentage of TUNEL-positive tumour cells in the light microscopic fields at × 400. Necrotic and inflammatory areas were excluded.

### Statistical analysis

All statistical analyses were carried out using commercially available software system (SPSS version 8.0, USA). All data are presented as mean ± standard deviation. The χ^2^-test and the Student's *t*-test were used to examine the association between Id-1 expression and the various clinicopathologic characteristics including PI, AI and MVD. The Kaplan–Meier method was used to calculate survival curves, and the log-rank test was performed to compare the difference in survival rates among the patient groups. A difference of *P*<0.05 between the groups was considered significant.

## RESULTS

### Id-1 overexpression in human primary pancreatic cancer

**Figure 1:**
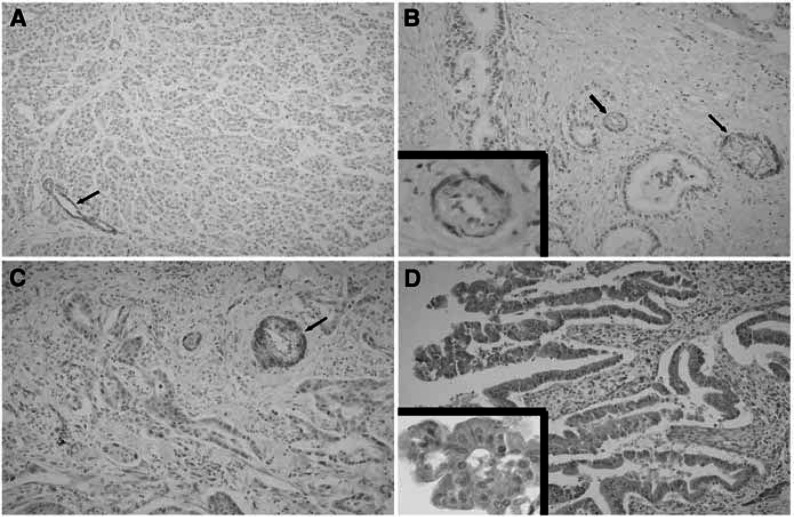
(**A**) Normal pancreatic tissue showing weak and focal staining for Id-1 (score 2). (**B**) The representative section showing Id-1expression absent in pancreatic cancer. Inset: In the blood vessel as a positive control, Id-1 expression is weak or negative in endothelial cells, while smooth muscle cells show strong immunoreactivity (× 400). (**C**, **D**) The representative sections showing moderate and strong Id-1 expression in pancreatic cancer, respectively. Inset: tumour cells show mostly diffuse cytoplasmic Id-1 expression (× 400). The arrows in each picture indicate the blood vessels used as an internal positive control.

All samples were stained for Id-1, and showed diffuse cytoplasmic staining from moderate to strong intensity in the tumour cells whereas the staining characteristics of normal pancreatic glands were diffuse and weak (Figure 1

). As shown in Table 1

**Table 1:** Relationships between Id-1 expression and the clinicopathologic characteristics of pancreatic adenocarcinomas

	**Id-1 expression**				
	**Score 2 (*n*=15)**	**Score 3 (*n*=18)**	**Score 4 (*n*=20)**	**Score 5 (*n*=12)**	***P*-value**
Age (years)	60.8±8.7	63.3±8.5	57.5±11.2	60.1±12.0	0.38
Male/female	9/6	14/4	11/9	6/6	0.38
Bilirubin	6.1±6.3	9.0±9.9	3.7±7.8	7.4±7.3	0.24
CA19-9	1006±1423	2550±5765	1580±2850	948±1864	0.59
Tumour size (cm)	3.1±2.9	4.7±3.4	3.1±1.1	3.7±1.8	0.38
*Location*					
Head	11	10	15	8	
Body/tail	4	8	5	4	0.58
*Tumour stage*					
I	3	1	6	4	
II	4	6	5	5	
III	3	3	3	1	
IV	5	8	6	2	0.66
*Metastasis*					
Positive	5	8	6	2	
Negative	10	10	14	10	0.46
*Differentiation*					
Well	3	1	2	2	
Moderated	11	13	16	9	
Poorly	0	4	2	1	0.46

All data are presented as the mean±s.d.

, all of the positive cases were classified by summing area and intensity scores: score 2, 15 cases (23.1%): score 3, 18 cases (27.7%): score 4, 20 cases (30.8%): score 5, 12 cases (18.5%). The average score of normal pancreatic tissue was 2.0±0.4. Thus, tumours with scores of 4 and 5 were considered to overexpress Id-1. In all, 32 cases (49.3%) revealed Id-1 overexpression *vs* normal tissue. No significant relationship was found between Id-1 expression and other clinicopathologic characteristics including age, bilirubin, tumour marker (CA 19-9), location, size, AJCC stage, differentiation and distant metastasis (Table 1).

### Id-1 overexpression is significantly related to angiogenesis but is not associated with cell proliferation

To investigate the association between Id-1 expression and cell proliferation or tumour angiogenesis, we examined PI, AI and MVD in tumour cells using Ki-67, TUNNEL and CD34 staining, respectively. As shown in Table 2

**Table 2:** Relationships between Id-1 expression and proliferation index, apoptotic index and microvessel density

	**Id-1 expression**				
	**Score 2 (*n*=15)**	**Score 3 (*n*=18)**	**Score 4 (*n*=20)**	**Score 5 (*n*=12)**	***P*-value**
MVD	71.1±34.1	58.3±21.5	109.5±58.0	110.2±57.2	0.002
PI	17.5±8.8	18.8±9.8	13.8±13.9	13.8±11.3	0.47
AI	0.95±0.87	0.64±0.52	0.88±0.94	0.97±0.84	0.62

**Figure 2:**
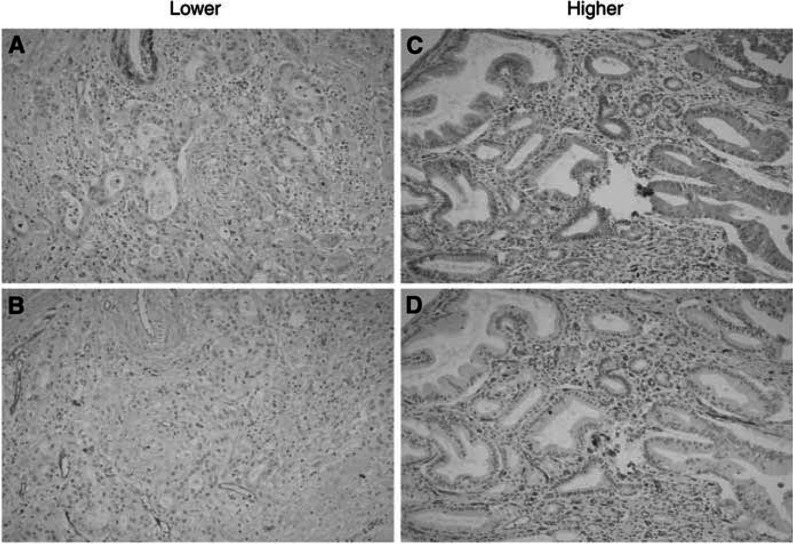
The representative sections showing the positive relation between of Id-1 expression and tumour angiogenesis (microvessel density) in pancreas cancers using immunohistochemical staining with Id-1 and CD34 antibodies. (**A**, **B**) Id-1 and CD 34 staining, respectively, in the tumour with lower expression (score 3). (**C**, **D**) Id-1 and CD 34 staining in the tumour with higher expression (score 5), respectively. Id-1 expression was found to be positively related with tumour angiogenesis (higher microvessel density).

All data are presented as the mean±s.d. MVD=microvessel density; PI=proliferation index (Ki-67 stain), AI=apoptotic index (TUNNEL stain).

, Id-1 expression was found to be significantly related with tumour MVD (*P*=0.002). In further analysis of subgroups showing higher and lower Id-1 expression, tumours with higher Id-1 expression (scores 4 and 5) also showed significantly higher MVD than tumours with lower Id-1 expression (scores 2 and 3) (111.18±57.14 *vs* 64.13±28.19, *P*<0.001) (Figures 2

and 3

**Figure 3:**
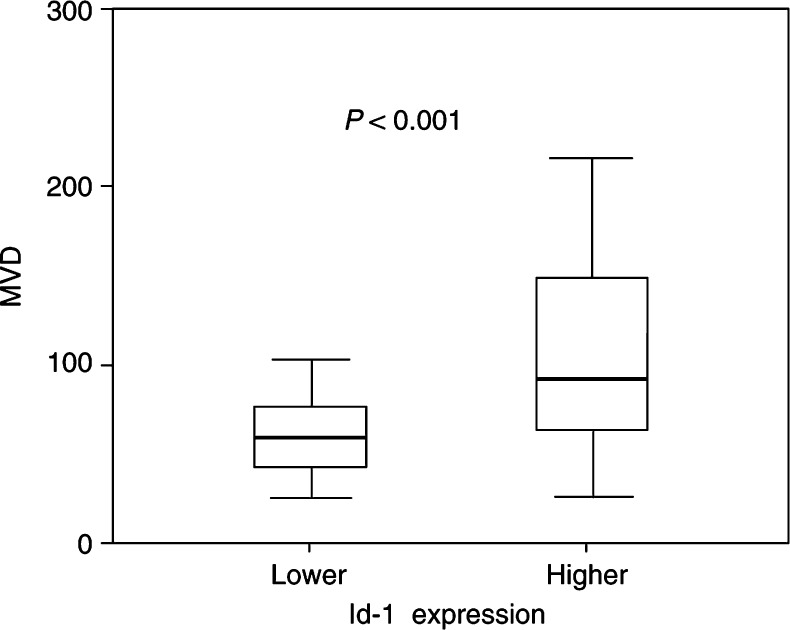
Analysis of subgroups with higher or lower Id-1 expressions. Tumours with higher Id-1 expression (scores 4 and 5) showed significantly higher MVD than tumours with lower Id-1 expression (scores 2 and 3) (111.18±57.14 *vs* 64.13± 28.19, *P*<0.001; Student's *t*-test).

). However, no significant relation was found between Id-1 expression and PI (tumour cell proliferation) or AI (tumour cell apoptosis) (*P*=0.47, 0.62, respectively) (Table 2). These results indicate that Id-1 is significantly associated with tumour angiogenesis in human pancreatic cancer, but that it is not related with tumour growth via enhanced cell proliferation or apoptotic inhibition.

### Id-1 overexpression is not significantly related with a poorer survival rate

**Figure 4:**
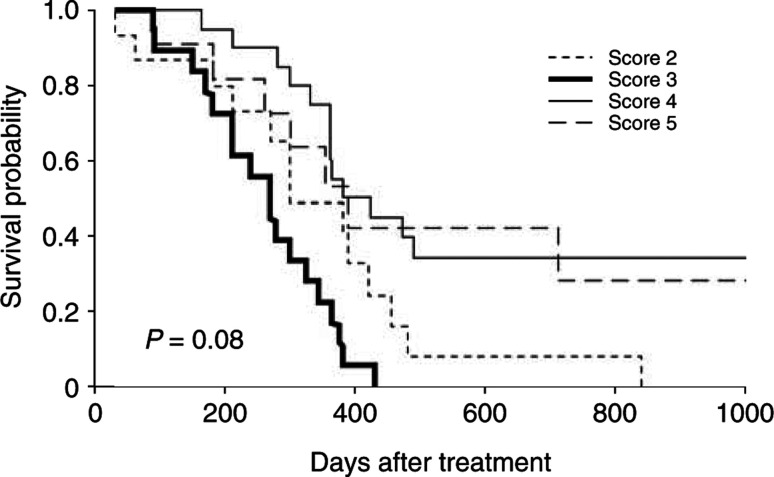
Actuarial survival rates according to the Id-1 expression in the pancreatic cancer patients (Kaplan–Meier method with log-rank test). Id-1 expression was not found to be significantly related with prognosis (*P*=0.08).

As a positive association of Id-1 expression with tumour angiogenesis, we examined the effect of Id-1 expression upon clinicopathologic prognostic factors in pancreatic cancer patients. As shown in Figure 4

, Kaplan–Meier survival curves showed no significant correlation between patient survival and Id-1 expression (*P*=0.08 by log-rank test). Therefore, these results suggest that Id-1 overexpression is not an significant indicator of a poor prognosis in human pancreatic cancer.

## DISCUSSION

Id protein is a family member of HLH transcription factors lacking DNA binding domain that has been shown to be an important regulator of cellular differentiation and proliferation (Norton, 2000). Id-1 overexpression has been reported in several human primary cancers including pancreatic, breast and cervical cancers. Evidence indicates that Id-1 may be associated with an aggressive phenotype of human cancers (Maruyama *et al*, 1999; Langlands *et al*, 2000; Lin *et al*, 2000; Polsky *et al*, 2001; Schindl *et al*, 2001; Ouyang *et al*, 2002a; Wang *et al*, 2002). In the present study, we observed Id-1 overexpression in human pancreatic cancer and we also investigated the association between its expression and cell proliferation and apoptotic indices, tumour MVD, and clinicopathological prognostic factors in an effort to clarify its biologic role in human pancreatic cancers. Our data show that Id-1 expression is significantly related with tumour MVD. However, it was not found to affect PI or AI, which are both important markers in cell cycle kinetics. Furthermore, no significant correlation was observed between Id-1 expression and the survival rate in pancreatic cancer patients. Our data indicate that Id-1 expression is closely related with tumour angiogenesis, but that it is not related to a poorer prognostic marker in human pancreatic cancers.

In the present study, Id-1 overexpression was found to be significantly associated with intratumoral microvessel proliferation, indicating that Id-1 has a significant role in tumour angiogenesis. Various molecules including VEGF, HIF-1 and Tie-2 receptors have been implicated with the angiogenesis (Risau, 1997). Lyden *et al* (1999) found that Id-1 protein is required for the proliferation and invasion of endothelial cells during angiogenesis. Moreover, loss of Id-1 and Id-3 also led to an unexpected defect in angiogenesis in knockout mice. Furthermore, the overexpression of Id-1 was shown to induce the expression of 120 kDa gelatinase in breast epithelial cells, which show an increase of invasiveness (Desprez *et al*, 1998). Therefore, taken together with previous studies, our data indicate that Id-1 is an important molecule in tumour angiogenesis and in the aggressiveness of human cancers. This is the first evidence that clarifies the association between Id-1 expression and tumour angiogenesis in human primary cancers. It appears that Id-1 protein may be an important new target molecule for antiangiogenic drug design in cancer treatment.

Several investigations have demonstrated that Id protein affects cell growth via the mechanisms of cell proliferation and/or apoptosis (Kim *et al*, 1999; Lin *et al*, 1999; Norton, 2000; Alani *et al*, 2001). It has been suggested that Id-1 and Id-2 proteins may function in the bHLH-mediated inactivation of the cyclin-dependent kinase inhibitors, for example, p21^WAF1^, p16^INK4a^ and p27^KIP1^ (Prabhu *et al*, 1997; Polsky *et al*, 2001; Ouyang *et al*, 2002b; Singh *et al*, 2002). In addition, Id-1 expression has been observed in small proliferating ducts and in the large ducts without dysplastic changes of chronic pancreatitis, which again indicates the proliferative potential of Id-1 (Maruyama *et al*, 1999). Thus, in the present study, we examined the proliferative potential of Id-1 expression in human primary pancreatic cancer by investigating the relation between its expression and the cell kinetic indices of PI and AI. However, our data show no significant relation between Id-1 expression and tumour cell proliferation in human primary pancreatic cancers. Therefore, it remains unclear whether Id-1 has a proliferative potential in human primary cancers although Id-1 has been implicated in the modulation of several cell cycle regulators. However, its proliferative potential in other human primary cancers remains to be clarified.

Id-1 plays a role in the early step of human carcinogenesis, for example, in melanoma and cervical cancer, whereas id-1 expression is markedly different between *in situ* and invasive breast carcinoma (Lin *et al*, 2000; Polsky *et al*, 2001; Schindl *et al*, 2001). These previous studies suggest that the role of Id-1 protein in human carcinogenesis is tissue specific. Id-1 was also found to be overexpressed in pancreatic cancers and in dysplastic/metaplastic ducts in chronic pancreatitis (Maruyama *et al*, 1999). Thus, it has been suggested that Id-1 is one of the early markers in pancreatic malignant transformation and that it may contribute to the early step carcinogenesis of human pancreatic cancer. In several human solid tumours, Id-1 overexpression has been reported to be an independent prognostic marker (Schindl *et al*, 2001,^2003^; Schoppmann *et al*, 2003). However, our data show that no significance between Id-1 overexpression and the clinicopathological biomarkers, such as survival rate, stage and tumour grade although Id-1 overexpression is related with tumour angiogenesis in pancreatic cancers. There are a number of possible explanations for this discrepancy concerning the Id-1 role in the prognoses between pancreatic cancer and other human cancers, such as cervical and breast cancers. Because pancreatic cancer rapidly progresses and is one of the most fatal malignant tumours, there is no reliable prognostic marker except at the tumour stage. Although it has been shown that human solid tumours have heterogeneous MVD, comparatively higher counts at the invasive edges of the tumours, and MVD is related with a poorer prognosis, there is still controversy on the prognostic significance of MVD in human pancreatic cancer because of its miserable clinicobiological course (Ellis *et al*, 1998; Khan *et al*, 2002; Gasparini, 1997). The other possible explanation is that Id-1 expression is tissue specific and has a different role in the development of various tissues that may contribute to different roles in carcinogenesis and in different biologic behaviours of different human cancers (Riechmann *et al*, 1994; Riechmann and Sablitzky, 1995; Lyden *et al*, 1999; Norton, 2000). Nevertheless, our results suggest that Id-1 overexpression does not have greater influence on the prognosis of human pancreatic cancer than other important prognostic markers, including stage and tumour grade.

In conclusion, our findings demonstrate the role of Id-1 overexpression in human primary pancreatic cancer. Id-1 overexpression was found to be closely related to tumour angiogenesis, a higher density of intratumoral vessel, but not with a poorer survival or a higher cell proliferation potential in human pancreatic cancer. This provides evidence that clarifies the association between Id-1 overexpression and tumour angiogenesis in human primary cancers and indicates Id-1 protein may be an important new target molecule for antiangiogenic drug design in cancer treatment. Additional studies are required to determine whether Id-1 has a similar impact on tumour angiogenesis in other human cancers.
